# Lipoblastoma: a clinicopathologic review of 23 cases from a major tertiary care center plus detailed review of literature

**DOI:** 10.1186/s13104-018-3153-8

**Published:** 2018-01-17

**Authors:** Jamshid Abdul-Ghafar, Zubair Ahmad, Muhammad Usman Tariq, Naila Kayani, Nasir Uddin

**Affiliations:** 1 Department of Pathology and Laboratory Medicine, Aga Khan University Hospital, Karachi, Pakistan; 2 Department of Pathology and Laboratory Medicine, French Medical Institute for Mothers & Children (FMIC), Behind Kabul Medical University Aliabad, P.O. Box: 472, Kabul, Afghanistan

**Keywords:** Lipoblastoma, Adipocyte, Benign, Recurrence

## Abstract

**Objective:**

Lipoblastoma is a rare neoplasm that occurs mostly in infants and children. Although benign, it has a tendency for local recurrence.

**Results:**

Clinical and pathological features of 23 cases of lipoblastoma described. Patients’ age ranged from 8 months to 18 years with mean and median age 4.1 and 2.5 years, respectively. Male:female ratio was 2.8:1. Most common sites were lower extremities (9 cases), followed by abdominal cavity and retroperitoneum (4 cases), and scrotum/groin (3 cases). Grossly, 22 tumors were well circumscribed and multi nodular. All cases showed lobules composed of adipocytes and lipoblasts with intervening fibrous septa and fine vascular network. Myxoid change, capsule formation and septation were seen in all cases. Zonation was seen in 2 cases. Follow-up was available in 14 out of 23 patients. Of these, 13 were alive and free of disease with no evidence of any recurrent lesion. One patient with a mediastinal infiltrating lipoblastoma experienced 4 recurrences. Lipoblastoma is a benign adipocytic neoplasm of infants and young children. Correlation of clinical and histological features helps in reaching a correct diagnosis. Owing to a high recurrence rate following incomplete resection, a complete resection is essential. Prognosis is excellent after complete resection.

**Electronic supplementary material:**

The online version of this article (10.1186/s13104-018-3153-8) contains supplementary material, which is available to authorized users.

## Introduction

Lipoblastomas are rare benign usually encapsulated neoplasms of infants and children, more common in males with a tendency for local recurrence, first described as lipoblastic tumors in infants and children under 3 years composed of fetal adipose tissue [1–5].

Local recurrence is common, up to 80%, in incompletely resected tumors [6–8]. Complete surgical excision is essential to prevent recurrence. Grossly, pale yellow, myxoid cut surface with small cystic foci and microscopically, lobules composed of immature adipose tissue separated by fibrous septa and lipoblasts in different stages are seen with no atypia or mitoses, plexiform capillary network and mature adipose tissue are seen in the central part of the lobules [9]. Lipoblastomas arise from embryonic white fat as rapidly enlarging masses. Most common locations include extremities and trunk [10–15]. Lipoblastomas have been reported in head and neck [12, 13, 16–21], mediastinum, axilla, labia, vulva, abdomen, retroperitoneum, groin, inguinoscrotal, and gluteal region [14–16, 22–27]. Lipoblastomatosis is reserved for lesions which demonstrate infiltrative growth [2, 8, 11, 16, 20, 21].

We report 23 cases and this series is the largest from the Indian sub-continent. Our aim was to describe the clinicopathological features of lipoblastomas which are important in their accurate diagnosis and differentiation from histologically similar neoplasms. The follow up information will help us understand their behavior.

## Main text

### Materials and methods

We searched the surgical pathology database of the two collaborative tertiary care hospitals Aga Khan University Hospital, Karachi, Pakistan and French Medical Institute for Mothers and Children, Kabul, Afghanistan. We retrieved 23 cases diagnosed as “Lipoblastoma” between 2006 and 2016. Inclusion criteria were presence of admixture of adipocytes and lipoblasts, primitive mesenchymal cells, plexiform vascular network and fibrous septations. Cases in which expression of immunohistochemical (IHC) markers (S100, CD34 and Desmin) was incompatible with histological features were excluded. Patients above 20 years were not included. Existing co-morbidities such as congenital heart disease or diabetes mellitus led to exclusion. Demographic data including patients’ age, gender, presenting complaints, tumor location, size and gross appearance was available from pathology reports. Hematoxylin and eosin and immunohistochemical slides were reviewed for microscopic features. We looked for zonation, myxoid change, lobulation and IHC expression. Slides were stained with CD34 (Ready To Use [RTU] Monoclonal antibody, QBEnd 10 clone, Dako), S100 (RTU Polyclonal antibody, Dako) and Desmin (RTU Monoclonal antibody, D33 clone, Dako). Follow up data of patients treated at our two institutions was obtained from hospital medical records. Follow up duration was defined as the time interval between the date of diagnosis and date of last follow up. For patients treated at other hospitals, follow up data was obtained from the guardians through interview conducted via telephone. Interview questions were pre-designed and a proforma was filled for each case. Follow up data was obtained from 14 patients. As this was a descriptive cross-sectional study, no statistical tests were applied. However, descriptive statistics such as mean, standard deviation and median for quantitative data and percentages for qualitative data were calculated after the data was entered in SPSS version 19 software.

### Results

Patients’ ages ranged from 8 months to 18 years, mean and median age 4.1 and 2.5 years, respectively. 17 (74%) were males and 6 (26%) were females (Male:female ratio: 2.8:1).

All patients presented with progressively enlarging painless swelling. Most common locations were lower extremities (thigh 6, leg 1, foot 2) abdominal cavity/retroperitoneum (n = 4) and scrotum/groin (n = 3) (Table 1).

**Table 1:** Summary of clinicopathological findings (n = 23)

Serial #	Age	Gender	Tumor size (cm)	Tumor site
1	2–5 years	Female	13	Retroperitoneum
2	< 2 year	Male	12.5	Back
3	2–5 years	Male	4.5	Neck
4	2–5 years	Male	22	Scrotum
5	2–5 years	Male	7	Groin
6	< 2 year	Male	15	Axilla
7	6–12 years	Male	N/A	Neck, parotid region
8	2–5 years	Male	28	Retroperitoneum
9	< 2 year	Male	5.5	Scrotum
10	< 2 year	Male	10.5	Abdomen
11	< 2 year	Male	7.5	Mesentery
12	6–12 years	Male	8.5	Foot
13	6–12 years	Female	10	Foot heel
14	6–12 years	Male	11	Thigh
15	> 12 years	Female	21	Mediastinum
16	> 12 years	Male	10	Thigh
17	< 2 year	Male	9	Leg
18	< 2 year	Male	9	Upper back
19	< 2 year	Male	4.5	Thigh
20	< 2 year	Male	8	Thigh
21	2–5 years	Female	7	Arm
22	2–5 years	Female	5	Thigh
23	< 2 year	Female	9.5	Thigh

**Fig. 1:**
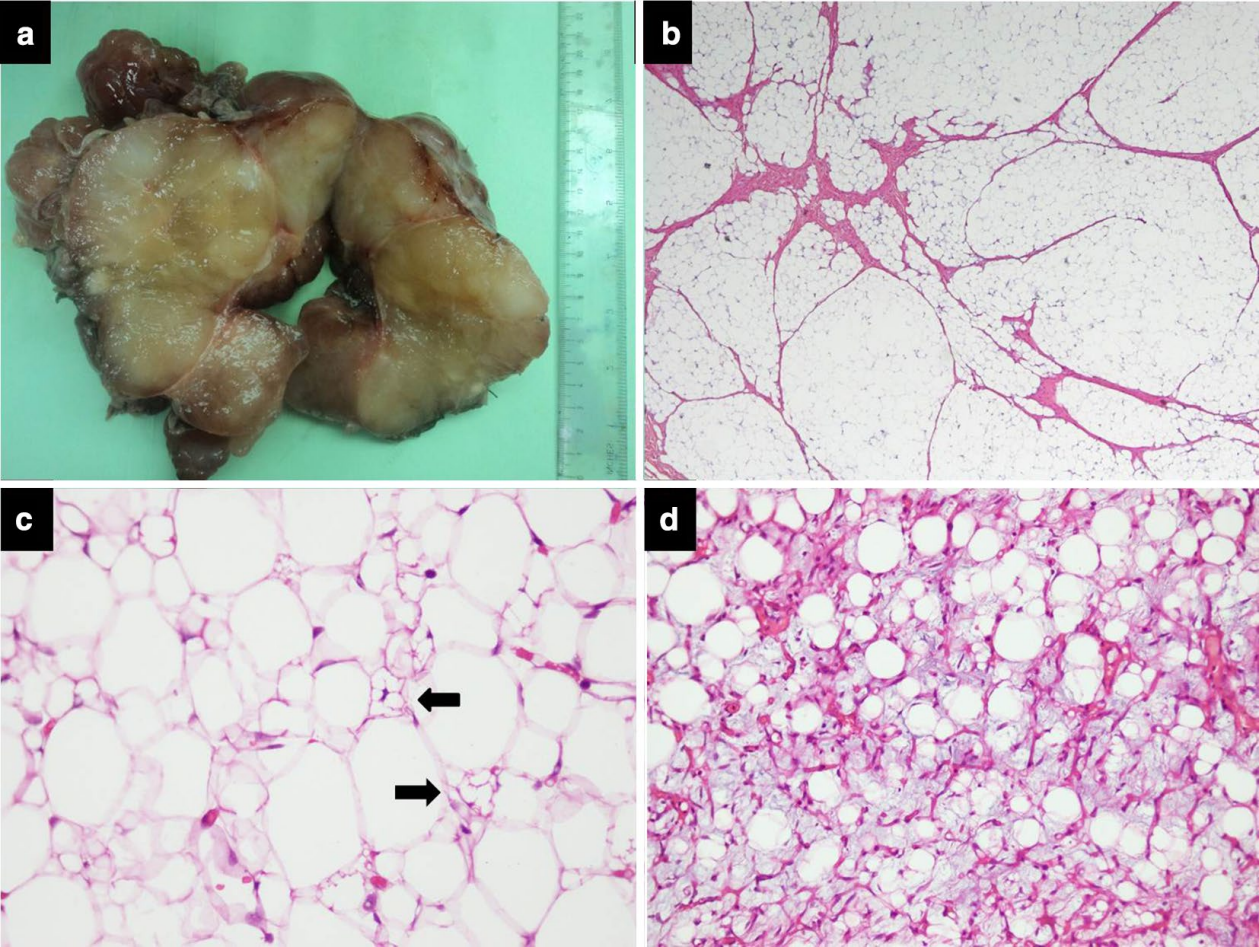
**a** Gross appearance of lipoblastoma. Tumor is encapsulated and cuut surface is yellow, soft and myxoid. Fibrous septations impart multinodular imparts appearance. **b** Microscopic features of of lipoblastoma. Fibrous septations divide the tumor into lobules. **c** Variable sized multivacuolated lipoblasts (arrows) admixed with adipocytes. **d** Thin walled branching (plexiform) vasculature

**Fig. 2:**
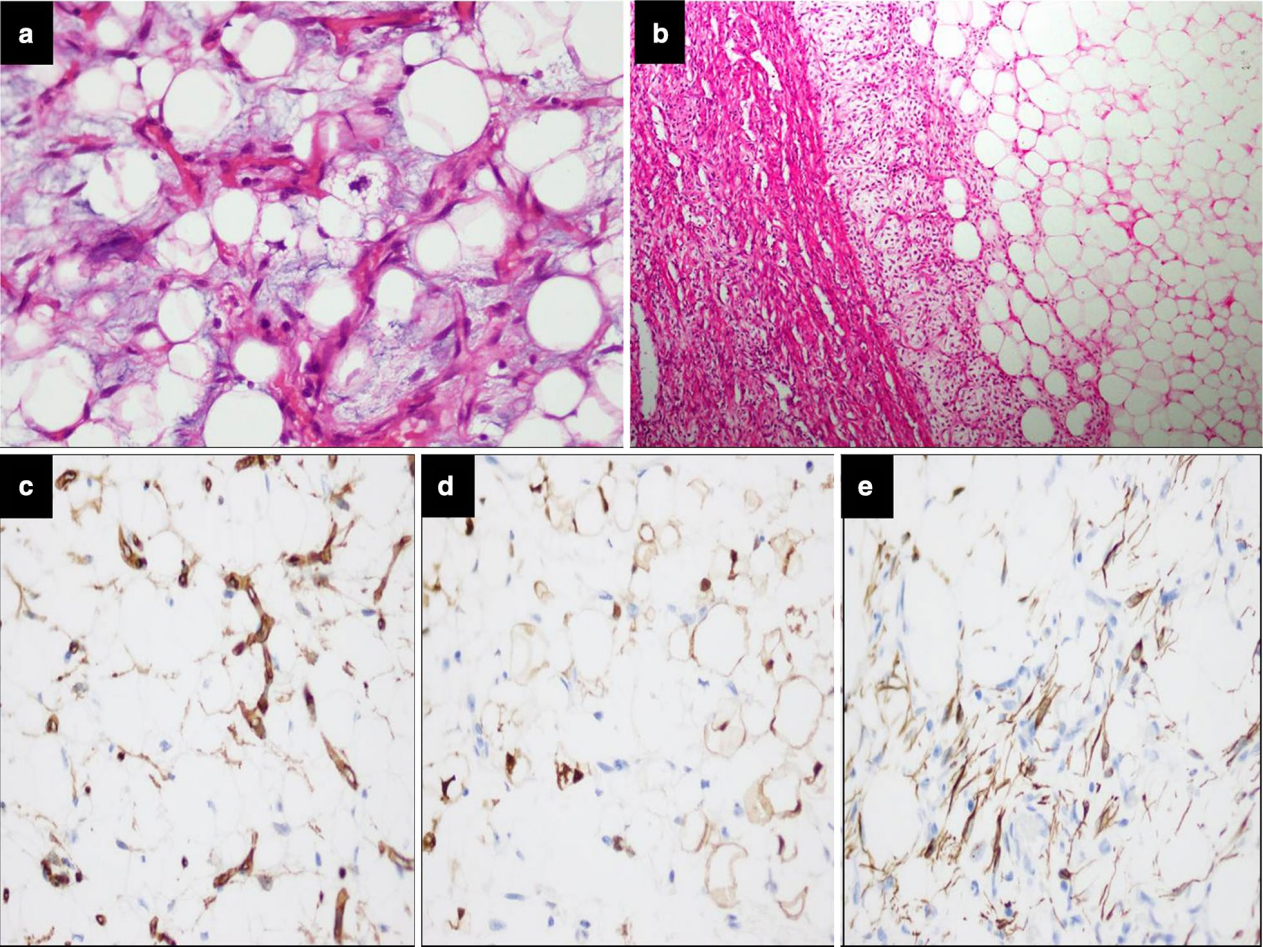
**a** Lipoblasts and plexiform vasculature is present against myxoid background. **b** Zonation; primitive mesenchymal cells at the edge of fibrous septa merging gradually with adipocytes. **c** Adipocytes and blood vessels demonstrating positive expression for CD34 IHC stain. **d** Adipocytes demonstrating positive expression for S100 IHC stain. **e** Primitive mesenchymal cells demonstrating positive expression for Desmin IHC stain

Grossly, 22 tumors were circumscribed and multinodular (Fig. 1a). One case had infiltrative margins. Tumor size ranged from 4.5 to 21 cm (mean, 10.8; median, 9.2 cm). Histologically, all showed lobules composed of adipocytes and lipoblasts separated by fibrous septa. A fine vascular network was seen. No nuclear atypia or mitoses was seen (Fig. 1b–d). Focal or diffuse myxoid change, encapsulation and septation were seen in all cases. Lobulation was seen in 19 (82.6%) and zonation in 2 cases (8.6%) (Fig. 2a, b).

Immunohistochemically, primitive mesenchymal cells were reactive for Desmin; adipocytes and vascular endothelial cells demonstrated positivity for CD34; adipocytes were positive for S100 protein (Fig. 2c–e).

Follow-up data was available in 14 patients. 13 patients were alive and well with no evidence of recurrence. Only patient with recurrence was a 14-year-old female with a mediastinal lipoblastoma with infiltrative margins who had multiple local recurrences at 17, 24, 45 and 47 months after surgery (Additional file 1: Table S1). The remaining 9 patients received treatment at other institutions and their medical records were unavailable.

### Discussion

Lipoblastomas are the second commonest pediatric adipocytic tumors after lipomas. Magnetic resonance imaging is the modality of choice for evaluation of tumor extension and planning of excision [11, 15–17, 19, 26–28]. Majority are 5 cm or less in size. However, larger tumors have been reported. Majority of tumors in our study were greater than 5 cm in size, mean size was 10.8 cm. This may be related to late presentation owing to poor socio-economic conditions and health facilities. Grossly, these are soft, lobulated and yellow, white or tan in color. Fat, myxoid nodules or cystic spaces separated by fine, white, fibrous septa are seen on cut surface [9, 14–17, 19, 21, 22, 24, 27, 29]. All tumors in our study were encapsulated, soft and showed fibrous septations. Our findings were similar to those reported in various published studies. Histologically, characteristic lobular architecture is seen with sheets of adipocytes separated by fibrovascular septa. The adipocytes demonstrate different stages of maturation from primitive spindle cells to vacuolated lipoblasts to mature adipocytes in varying proportions. Myxoid areas with primitive mesenchymal cells and plexiform capillary network are seen. The lobules show a zonal pattern of maturation with mature adipocytes in the center and myxoid areas at the periphery. A greater proportion of mature adipocytes (lipomatous pattern) are an indication of maturation. Variable numbers of residual lipoblasts are seen in less mature myxoid areas [1, 2, 7, 9, 16, 24, 27]. Our findings were similar to those reported in various studies. We did not find any association between maturity and increasing age of patient nor between maturation and tumor site. In one study [16], lobules bordered by septa were seen in 44%, prominent blood vessels in 76% and myxoid foci in 52% cases. Mitoses are rare, abnormal mitoses are never seen [9, 16, 17, 19–21, 23, 29]. On IHC, adipocytes are positive for CD34 and S100 protein, while primitive cells are positive for Desmin [9]. Our findings were in concordance with the reported findings.

The genetic hallmark are clonal rearrangements involving chromosomal region 8q11 > q13 (8q12). The oncogene PLAG1 (pleomorphic adenoma gene 1) is located on band 8q12. PLAG1 is activated by a breakpoint in the 8q11–13 region or polysomy of chromosome 8 [10, 11, 23, 25, 28–32]. Molecular analysis is important in the accurate diagnosis and optimum management.

The differential diagnoses include pediatric lipoma, hibernoma, pediatric myxoid liposarcoma (ML) and well differentiated liposarcoma (WDL). Lipoblasts or primitive mesenchymal cells are not seen in lipomas while hibernomas are composed of brown fat cells which have a central nucleus and abundant finely granular cytoplasm. Lipomas are not common in extremities and do not show chromosome 8 abnormalities. MLs are very rare in children under 10 and are always deeply situated; lipoblastomas may be superficial or deep. MLs show a characteristic ‘pulmonary edema’ pattern, which is attributable to pools of stromal mucin. This is not seen in lipoblastomas. MLs may have hypercellular areas while lipoblastomas are uniformly hypocellular. MLs show nuclear atypia and do not show the lobulated growth pattern of lipoblastomas. In MLs, maturation occurs towards the periphery while in lipoblastomas, maturation occurs towards the center of the lobules, periphery is composed of more primitive cells. MLs do not show chromosomal 8 rearrangements. They are characterized in 95% cases by recurrent translocation t (12;16) q (13; p11) which results in fusion of FUS-DDIT3 genes. It is important to differentiate between lipoblastoma and ML as the latter are malignant and require aggressive treatment. Immunohistochemically, liposarcomas are MDM2 and CDK4 positive while lipoblastomas are negative. The majority of ML and WDL are positive for p16 while large majority of lipoblastomas are negative. A negative p16 staining may be helpful in excluding liposarcoma in cases occurring in adolescence or those with late recurrences [33]. Chromosome 8 abnormalities are not seen in WDL.

The treatment of choice is complete surgical resection with preservation of vital organs. Prognosis is excellent after complete excision even in large tumors. Recurrence rates vary from 13 to 46% depending on extent of resection. Clinical outcome depends on completeness of resection with a high recurrence rate in incompletely resected tumors. Close follow up for minimum of 5 years is recommended. Various studies have not reported any recurrences in completely excised tumors with follow ups of 2 months–10 years and median follow up times ranging from 38 to 42 months [11, 14, 15, 19, 22, 24, 25, 27, 30].

In our study, 74% patients were males. Male predominance was noted in multiple studies [1, 2, 5, 6, 8, 24, 27]. In our series, age range was 8 months–18 years, 11 (47.8%) patients were younger than 3 years of age, 16 (69.6%) were 5 years or younger and 19 (82.6%) were 10 years or younger. In Coffin et al.’s series [1], about 90% patients were 10 years or younger. In another series [16], 84% patients were younger than 5 years. In various studies [1, 24, 27], ages ranged from 3 months to 16 years.

Most common locations include extremities and trunk [10–15]. Ten cases (43.5%) in our study were located in the extremities, thigh was the commonest location (26%). In one study, 41% were located in the lower extremities while 10% were located in the trunk [34]. In another study, 44% were located in the extremities [16]. As many as 64% cases in one review were located in the trunk [1].

22 cases were discrete with intact capsules. In contrast, only 44% tumors reviewed by Collins and Chatten [16] were discrete while 56% had irregular margins. Head and neck was a common location (16,18). In our series, 2 (8.6%) cases were located in the head and neck.

All patients presented with progressively enlarging painless swelling, the commonest complaint [16, 24, 27] seen in up to 92% cases.

Tumor size ranged from 4.5 to 21 cm, mean and median size, 10.8 and 9.2 cm, larger compared to average reported size (5 cm or less). 16 (69.6%) cases were larger than 5 while 10 (43.5%) were larger than 10 cm. In one study [16], 60% measured less than 5 cm. Various studies reported tumor sizes between 5 and 15 cm [24, 27]. The largest reported case measured 26 × 21 cm [35]. Grossly, all cases showed lobulated appearance, were soft in consistency and had a yellowish and myxoid cut surface. One case had infiltrative margins.

IHC (CD34, S100 protein and Desmin) was performed in all cases, often retrospectively when the authors reviewed the slides. We recommend routine IHC staining in all pediatric lipomatous tumors since these help in accurate diagnosis especially in cases with equivocal morphologic features. Follow up was available in 14 cases (Additional file 1: Table S1). Follow up period ranged from 1 to 109 months, median time of follow up was 12 months. Only 1 patient, a 14-year-old girl with a mediastinal lipoblastoma developed 4 separate recurrences at 17, 24, 45 and 47 months and underwent repeated resections. The remaining 13 were alive and free of disease with no recurrences. A number of studies did not report any recurrence during follow up periods of 1–2 years [17, 19], a case of inguinolabial lipoblastoma did not recur after 6 years of follow up [22]. A low rate of recurrence was reported by Fallon et al. [32]. Two recent studies [24, 27] reported no recurrences in any of their patients with median follow up of 42 months (range 18–84 months) and 38 months (range 1.8 months–10 years) respectively.

Complete surgical resection and follow up for minimum of 5 years is essential. It is hoped that this series will help in increasing awareness in our region about these rare tumors among surgeons and pathologists. Pathology departments will be encouraged to develop protocols regarding use of various IHC stains in accurate diagnosis of pediatric adipocytic neoplasms. Correlation between clinical and histological features helps in reaching an accurate diagnosis. Confirmation by molecular studies is recommended.

## Limitations

The main limitation of our study was small sample size (23 cases) but it needs to be emphasized that lipoblastomas are rare tumors, these 23 cases comprise all cases diagnosed in our two institutions over a 10 year period and represent the largest series on these rare tumors from this region. Absence of follow up in some cases was another limitation and was mainly because in many remote regions of both countries, communication facilities are suboptimal.

## Additional file

**Additional file 1: Table S1.** Follow up of patients (n = 14).
