# Supplementary material for: Lipoblastoma: a clinicopathologic review of 23 cases from a major tertiary care center plus detailed review of literature
Source: BMC Res Notes. 2018 Jan 17;11:42. doi: 10.1186/s13104-018-3153-8 (PMC5773143; doi:10.1186/s13104-018-3153-8)
Supplement: Supplementary file 1 — Additional file 1: Table S1. Follow up of patients (n = 14). [file 13104_2018_3153_MOESM1_ESM.docx]

**Table S1. Follow up of patients (n=14)**

| **No.** | **Patients’ age at diagnosis** | **Year of resection** | **Follow up duration (months)** | **Outcome** |
| --- | --- | --- | --- | --- |
| 1 | <2 years | 2008 | 109 | Alive and disease free |
| 2 | >12 years | 2010 | 79 | Alive with 4 Recurrences at 17,24,45 and 47 months |
| 3 | 2-5 years | 2011 | 72 | Alive and disease free |
| 4 | 2-5 years | 2011 | 70 | Alive and disease free |
| 5 | 2-5 years | 2014 | 37 | Alive and disease free |
| 6 | 6-12 years | 2015 | 21 | Alive and disease free |
| 7 | 6-12 years | 2015 | 24 | Alive and disease free |
| 8 | <2 years | 2016 | 14 | Alive and disease free |
| 9 | <2 years | 2016 | 14 | Alive and disease free |
| 10 | <2 years | 2017 | 6 | Alive and disease free |
| 11 | 2-5 years | 2017 | 4 | Alive and disease free |
| 12 | <2 years | 2017 | 3 | Alive and disease free |
| 13 | <2 years | 2017 | 1 | Alive and disease free |
| 14 | 2-5 years | 2017 | 1 | Alive and disease free |
